# Effectiveness of Massage Therapy and Abdominal Hypopressive Gymnastics in Nonspecific Chronic Low Back Pain: A Randomized Controlled Pilot Study

**DOI:** 10.1155/2018/3684194

**Published:** 2018-02-22

**Authors:** L. Bellido-Fernández, J. J. Jiménez-Rejano, R. Chillón-Martínez, M. A. Gómez-Benítez, M. De-La-Casa-Almeida, M. Rebollo-Salas

**Affiliations:** ^1^Physiotherapy Department, Faculty of Nursing, Physiotherapy and Podiatry, University of Seville, C/ Avicena S/N, 41009 Seville, Spain; ^2^Podiatry Department, University of Seville, C/ Avicena S/N, 41009 Seville, Spain

## Abstract

**Background:**

There are a great number of interventions in physiotherapy, but with little evidence of their effectiveness in chronic low back pain. Therefore, this study assesses effectiveness of Massage Therapy and Abdominal Hypopressive Gymnastics and the combination of both to decrease pain and lumbar disability while increasing joint mobility and quality of life in patients with chronic nonspecific low back pain.

**Methods:**

A randomized, single-blinded, controlled, clinical trial with sample (*n* = 27) was comprised of patients between 20 and 65 years, diagnosed with pain of mechanical origin characterized by having a duration of at least 12 weeks and no serious complications. Each group received 8 interventions of 30 minutes.

**Results:**

Friedman ANOVA test obtained statistically significant differences of Oswestry, NRS, and Schober variables (*p* < 0.05) in the three measurements (pretest, posttest 1, and posttest 2), in each individual group. ANOVA Kruskal-Wallis test was used for comparison between groups, and Oswestry Disability values were significantly higher (*p* = 0.024) in the group receiving both treatments.

**Conclusion:**

Both individual groups reduce pain levels, improve disability, and increase the flexibility of the lumbar spine. The combination therapy provides greater benefits in terms of lumbar disability. This study is registered on March 8, 2016, with NCT02721914.

## 1. Introduction

Chronic low back pain has been and is one of the dilemmas in the field of health in the XXI century. This is due to its high incidence in our current society; it is also one of the most disabling pathologies affecting young [1–3] adults, most of whom, up to 90%, are diagnosed with nonspecific [4–6] low back pain; and between 2% and 7% will eventually suffer chronic pain which will interfere with the individual functional abilities, thus affecting their everyday life.

Physiotherapy uses a great number of interventions whose goal is the treatment and functional recovery of the population affected by nonspecific low back pain [7, 8].* Massage Therapy* is one of the oldest types and with better results [9], reduces pain level, and improves the capability of the individual who suffers from this pathology [10]. It has great benefits for health [11] and improves the circulatory, muscular, and nervous system [12]. The Massage Therapy is a method of global body balance and relaxation [13].

Therapeutic exercise, also controversial, is currently gaining ground regarding effectiveness in clinical and scientific practice [14, 15]. There are many approaches concerning active recovery [16], but there is no clear evidence of a specific protocol [17].

The training of the muscles which give stability to the trunk [18], as well as that of the pelvic floor muscles, helps improve the often cited low back pain [19–22]. One of the outstanding techniques is the* Abdominal Hypopressive Gymnastics* which is becoming increasingly popular. It is about postural exercises, which allow a decrease in pressure in the abdominal, perineal [23], and thoracic cavities [24, 25]. The hypopressive exercise produces the direct activation of the transverse abdominal muscle, which allows strengthening the abdominal girdle and stabilizing the spine [24]. It provides benefits such as strengthening the abdominal muscles, making the lumbar spine and the hamstring muscles more flexible, and rearranging the body posture [26–28].

The need to know the effects of both Massage Therapy and Abdominal Hypopressive Gymnastics to a greater depth marks the starting point to continue working and try to improve the different guidelines and protocols of intervention.

Therefore, the purpose of this study is to determine whether the Massage Therapy and the Abdominal Hypopressive Gymnastics (or the combination of both procedures) have effect or impact on the quality of life and level of health of patients with nonspecific low back pain.

## 2. Material and Method

### 2.1. Design Type and Sample

This is a controlled randomized clinical trial, with three groups in parallel. It was developed in the facilities belonging to the School of Nursing, Physiotherapy and Podiatry of the University of Seville. It lasted 8 weeks, between April and June 2016. Prior to this work, a favorable decision was obtained from the Research Ethics Committee of the Virgen Macarena University Hospital Center.

The patients who composed the sample came from traumatology department of the Back School of the University of Seville. All selected subjects were diagnosed with nonspecific low back pain; likewise all of them had to meet the inclusion criteria and without showing any of the exclusion criteria. Written and verbal information were given to all the subjects through informed consent; and once it was signed they became a part of the study.

**Figure 1:**
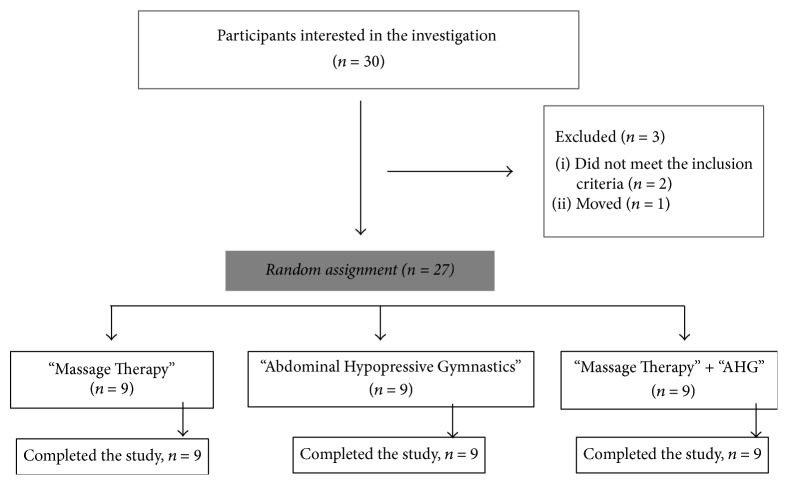
Diagram of the participants.

The sample consisted of 27 subjects with a mean age of 32.59 years (standard deviation) and comprised 23 women and 4 males, who were divided into three groups in a random manner (27 ballots in an opaque container) in group 1 Massage Therapy (*n* = 9), group 2 AHG (*n* = 9), and group 3 Massage Therapy + AHG (*n* = 9) (Figure 1). The sample was composed of nonconsecutive probabilistic sampling and all participants were selected by convenience sampling.

In line with other studies such as Miranda et al. [29], Caufriez et al. [27], or Stieglitz et al. [30], we start this study with a small sample size. This will help us in future research to calculate sample size in the following clinical trial.

### 2.2. Inclusion and Exclusion Criteria

Patients of both sexes, aged between 20 and 65 years, diagnosed with chronic nonspecific low back pain, with mechanical pain having a duration of at least 12 weeks and not presenting severe complications were included in this study. The exclusion criteria were diagnosis of arterial hypertension, progressive neurological deficit, pregnancy or suspected pregnancy, and being under pharmacological or psychiatric treatment.

### 2.3. Measurement Instruments and Intervention Protocol

Different scales and questionnaires were employed to measure four main variables:

- *Pain Intensity* is measured using the* Numerical Rating Scale* (NRS) that goes from 0 = no pain to 10 = maximum pain. According to some researchers, it is a valid [31, 32] and reliable [33–35] tool at the clinical level and also in the assessment of pain induced at the experimental level [36]. In addition, it has been shown to be sensitive to the effects of treatments [32, 37, 38]. In relation to this, it seems that this scale is one of the most adequate types for pain assessment [38].
- *Functionality* is measured using the* Oswestry Disability Index* (0% = minimum functional disability; 100% = severe functional disability). This questionnaire is the most used and recommended worldwide [39]. Alcántara-Bumbiedro et al. [40] carried out the transcultural adaptation to the Spanish population in 1995 of the Oswestry questionnaire, proving to be valid and reliable and have an adequate internal consistency.
- *Quality of life* is assessed using the SF-12 questionnaire, a shortened version of the SF-36, which evaluates both the functional status and mental health. It is a scale transculturally adapted to Spanish [41] and consists of a subset of 12 items of the SF-36 obtained from multiple regression and has proved to be a useful version with which it is intended to measure the aspects of health and quality of life of patients [41, 42].
- *Lumbar flexibility* is measured using the Schober Test. It has been shown to have validity and reliability [43].

The registry of all these parameters, performed by the blinded external evaluator, was carried out on three occasions: initial evaluation (Pretest), midterm evaluation, in the middle of the treatment (posttest 1), four weeks after the start of treatment, and a final evaluation, which was made at the end of the interventions (posttest 2).

Study development that lasted a total of 5 weeks was characterized for having 8 interventions of 30 minutes each, excluding the learning time and time required for the different evaluations. The first 3 weeks, 2 weekly sessions were applied (distributed on Monday and Thursday or on Tuesday and Friday), and a weekly session was applied during the remaining two weeks.

**Figure 2:**
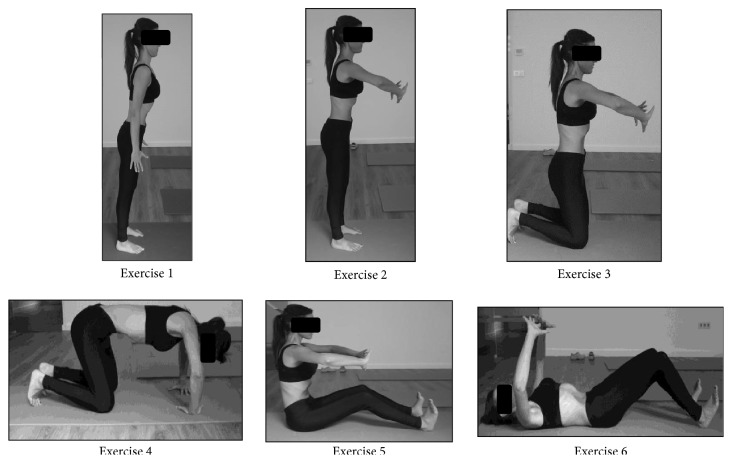
Abdominal Hypopressive Gymnastics protocol.

A single specialist physiotherapist performed the treatment that participants of each group received. The different interventions were distributed as follows:

- Group 1 received a Massage Therapy protocol focused on their spine, designed for the recovery of the thoracic-lumbar and cervical system, as well as that of the entire fascial system, taking the ergonomics basis of the physiotherapist into consideration [9, 44, 45]. So, the subjects of group 1 received a combination of structural massage combined with myofascial therapy [45].
- Group 2: they performed a series of 6 static abdominal hypopressive exercises (Figure 2); they repeated each exercise three times plus a previous phase of learning and a minimum rest to complete the series [27, 46].
- Group 3: having similar characteristics, this group received 4 interventions of Massage Therapy and another 4 of Abdominal Hypopressive Gymnastics, alternated, respectively.

### 2.4. Statistical Data Analysis

A blinded specialist in statistics (other than those responsible for the intervention, the random allocation, and data collection) was assigned to organize and analyze the data, using the SPSS version 22.0 statistical package and considering a confidence interval of 95% (*p* value < 0,05).

The effectiveness of the three applied interventions was examined by the intention-to-treat method, comparing the three groups (Group 1: Massage Therapy; Group 2: AHG; Group 3: Massage Therapy + AHG). The Shapiro-Wilk test was used to verify the normality of the sample and subsequently a descriptive data analysis was performed.

The one-way ANOVA test was used to verify the homogeneity of the three groups in terms of “age” and pretest of all the dependent variables and the “gender” variable with the chi-square test of Pearson (Table 1).

Subsequently, the differences among the measured variables were obtained between the three measurements as well as the comparison among the three groups, using the two-way ANOVA with the complementary tests.

## 3. Results

### 3.1. Homogeneity of the Groups

We found that the three groups are homogenous in terms of gender distribution, using the chi-square test of Pearson (*X*^2^(2,27) = 4,109, *p* = 0,128). And, by using the one-way ANOVA test, it was found that they are homogenous in terms of age and pretest of all the dependent variables (*p*< 0,05) (Table 1).

### 3.2. Effectiveness of Each Intervention

**Figure 3:**
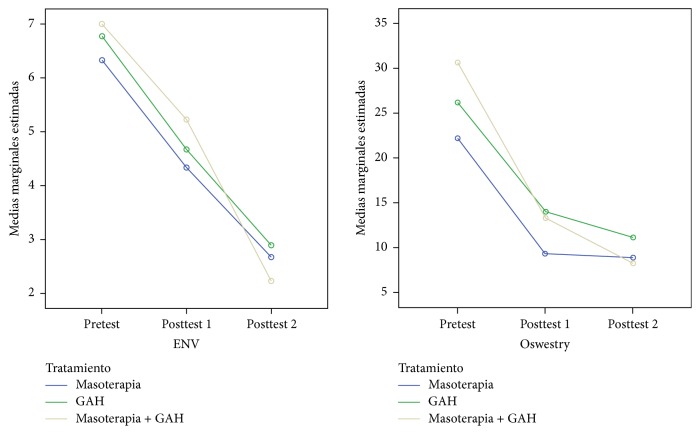
Marginal mean diagram of the Oswestry and NRS variable. The group receiving both treatments (Massage Therapy + AHG) obtained a greater statistically significant difference between pretest and posttest 2 against Massage Therapy group (*p* = 0,024).

Three main measures were made: pretest, posttest 1 (midterm measurement), and posttest 2 (at the end of treatment). The values of the means and standard deviations of each of these measurements in each of the three intervention groups are shown in Table 2. Significant statistical differences were obtained among the three measurements performed in the variables of the low back pain disability using the Oswestry questionnaire, NRS, and Schober's test (*p*< 0,001). No significant statistical differences were found among the three measurements (*p* = 0,148) using the SF-12 questionnaire (Figure 3).

### 3.3. Effectiveness of Each Treatment regarding the Others

The group that received both interventions (Massage Therapy + AHG) obtained significantly high values in difference 2 (*p* = 0,024), which is the disability variable measured using the Oswestry questionnaire (the difference between pretest and final posttreatment measurement), with respect to the group that only received Massage Therapy (Figure 3, Table 2).

## 4. Discussion

### 4.1. Intervention through Massage Therapy Protocols

*Massage Therapy* has proved to be the oldest therapy used, and thus it is one of the most studied therapies [47]. While* Cherkin* et al. [9] obtained significant and similar results in two types of Massage Therapy (structural and relaxing) in 10 treatment sessions,* Netchanok and his collaborators* [12] compared Thai and Swedish massage in their review and obtained similar results in terms of reducing pain and improving disability using the* Numerical Rating Scale* (NRS). However, they do not determine the protocol effectiveness according to the mode and duration of the interventions, a question that, in the current study, we try to delimit by adjusting a single protocol of Massage Therapy, performed by the same specialist physiotherapist in all cases.

Concerning other treatments, Massage Therapy has been prominent but not with great evidence, as in the case of* Furlan* et al. [44], whose review only highlights three clinical trials in which this protocol reduces pain levels with regard to the placebo; and it also reduces the level of disability compared to acupuncture. As in the review of* Kumar* et al. [48], with significant results in those cases comparing placebo to simple relaxing techniques, it was not clear whether it was the best option when compared to other manual therapy options. It is not included in the* American Physical Therapist Association Practice Guideline* [14], and, from our point of view, they should contemplate including it, after verifying the results that we obtained in the current study (Figure 3); moreover, its use in other studies [49] has proved that it improves sleep and reduces anxiety.

### 4.2. Intervention Using Abdominal Hypopressive Gymnastics

*Abdominal Hypopressive Gymnastics* is one of the procedures used to improve tissue mobilization and enhance a faster and more effective recovery of the injury [50]. There have been few studies in this research, in which AHG isolated is used for the chronic low back pain, although it has been used with healthy patients [24, 26, 27, 51] or with different pathologies [21, 52]. Only one study mentions the use of the Abdominal Hypopressive Gymnastics method for the chronic nonspecific low back pain [53]. After a total of 40 sessions of 40 minutes each, the group receiving AHG (*n* = 10) improved significantly in terms of lower limb flexibility (*p*< 0,05), as well as lumbar spine mobility, although it was not superior to the group receiving a different method. If we pay attention to the sample of our investigation (*n* = 9), we can see that there are significant statistical improvements regarding lumbar flexibility, immediately after the treatment concluded (Table 2). Compared to Galindo Torres and Espinoza [53] study, our data are closer to an effective result, since we carried out fewer treatment sessions and they lasted a shorter time.* Caufriez and collaborators* [27] show effectiveness in the body posture by increasing the trunk self-stretching and strengthening the paravertebral muscles, but not according to Schober's test values; this may be because they were subjects with a normal parameter in lumbar spine mobility. However, Rial et al. [26] study could observe significant differences in Schober's test with just one hypopressive session (*p*< 0,001) and in the fingertip-to-floor test with the subjects in this case being nondiagnosed pathology females.

Therefore, the AHG appears to have an impact on spine flexibility in both healthy subjects and chronic low back pain patients.

### 4.3. Intervention through Combined Massage Therapy and AHG Therapy and Comparison among Groups

We have not been able to find research studies, in which Massage Therapy and Abdominal Hypopressive Gymnastics have been combined in the therapeutic approach to chronic nonspecific low back pain with which we could compare our results.

Nevertheless, we have found a recent clinical study, published in 2014 by* Yang* et al. [54], in which they used a therapeutic massage known as Tui Na whose origin comes from traditional Chinese medicine and a series of core exercises (trunk and abdominals stabilizing exercises). Pain and functional disability are more significant in the group receiving combined therapy at the end of treatment. As opposed to its 8 weeks and a total of 40 sessions of therapeutic intervention, our results show the aforementioned significance in only 8 sessions of 30 minutes each. Besides, this group obtained a statistically significant improvement for Schober's test in only 4 sessions of Massage Therapy and AHG combined. However, Yang et al. [54] do not measure the impact on lumbar mobility.

By comparing groups (control versus experimental) we can observe how these authors get pain and functionality improvements in the experimental group. But they do not compare which one is more significant [54], whereas in our study the reduction of disability caused by lumbar pain (Oswestry difference) is more effective in the group receiving the combined treatment (Figure 3). In fact, this group showed a large size Cohen's effect *d* = 1.32 versus the manual therapy group. This effect size is very high and is within the range of values established as clinically relevant according to the authors Parker et al. [55].

The low levels of relapse measured by* Yang* et al. [54] provide encouraging results to use passive and active techniques, in the same treatment protocol, for pathologies such as chronic low back pain with a greater range of benefits. It is true that our study, even with high statistical significance in values such as pain, functionality, and movement, manages to measure the effects in a short time. But we consider that the wide number of interventions of Yang et al. study [54] can increase the costs of the rehabilitation proposal and as a result devalue the benefits measured in the long-term.

All the data obtained in this study represent an important advance, since the characteristics of the sample, as well as the selection process, allow extrapolating these results for the rest of the population.

### 4.4. Limitations of the Current Study

- The blinding of the physiotherapist responsible for applying the treatment was not possible given the characteristics of the research.
- Being a pilot study having a small sample, with lack of sample size calculation, its generalizability and applicability are difficult.
- There is a lack of standardized intervention parameters.
- In general more studies of this type will be necessary in order to achieve a greater level of evidence.

## 5. Conclusions

According to the results obtained and to the previously established goals of the current investigation, the conclusions reached are explained in detail as follows:

- The application of Massage Therapy in patients with chronic nonspecific low back pain could promote benefits in terms of the level of pain reduction, lumbar spine mobility, and disability improvement. This treatment turns out to be as effective as an* abdominal hypopressive exercises* program. No significant differences were observed concerning quality of life.
- *Combined Massage Therapy* treatment and* Abdominal Hypopressive Gymnastics* protocol applied in patients with chronic nonspecific low back pain could bring improvements in lumbar disability, could reduce of pain levels, and could increase flexibility of the lumbar spine in the short term. In addition, it turned out to be more effective, in the short term, in reducing the disability caused by low back pain than the application of a single Massage Therapy protocol.

## Figures and Tables

**Table 1:** Homogeneity of three study groups in the distribution by gender, age, and pretest of the dependent variables.

Variable	Group	Frequency	Percentage	Significance
Gender				
Male	Massage Therapy	3	33,3%	*p* = 0,128
	AHG	0	0%	
	Massage Therapy + AHG	1	11,1%	
Female	Massage Therapy	6	66,7%	
	AHG	9	100%	
	Massage Therapy + AHG	8	88,9%	

Variable	Group	Median	First and third quartiles	Significance

Age	Massage Therapy	26,00	23,50; 34,50	*p* = 0,712
	AHG	24,00	22,00; 32,00	
	Massage Therapy + AHG	36,67	20,50; 55,50	

Oswestry	Massage Therapy	22,00	16,00; 27,00	*p* = 0,132
	AHG	30,00	16,00; 35,00	
	Massage Therapy + AHG	34,00	24,00; 37,00	

NRS	Massage Therapy	7,00	5,50; 7,00	*p* = 0,722
	AHG	7,00	5,00; 8,50	
	Massage Therapy + AHG	7,00	6,00; 7,50	

Schober	Massage Therapy	5,93	5,41; 6,38	*p* = 0,253
	AHG	6,53	5,53; 6,94	
	Massage Therapy + AHG	5,83	5,21; 6,01	

SF-12	Massage Therapy	32,00	31,00; 34,00	*p* = 0,295
	AHG	31,00	28,00; 33,00	
	Massage Therapy + AHG	32,00	29,00; 34,00	

**Table 2:** Contrast among three groups of treatment.

Variable	Group	Measuring	Median	Q1 and Q3	Differences between pretest and posttest at the end of treatment (difference 2)		
					Median	Q1 and Q3	Significance
Oswestry	Massage Therapy	Pretest	22,00	16,00; 27,00	14,00	7,00; 20,00	*p* = 0,025
		Posttest 1	6,00	4,00; 15,00			
		Posttest 2	8,00	5,00; 14,00			
	AHG	Pretest	30,00	16,00; 35,00	18,00	6,00; 18,00	
		Posttest 1	14,00	11,00; 17,00			
		Posttest 2	12,00	8,00; 16,00			
	Massage + AHG	Pretest	34,00	24,00; 37,00	20,00	11,00; 24,00	
		Posttest 1	14,00	10,00; 15,00			
		Posttest 2	8,00	6,00; 11,00			

NRS	Massage Therapy	Pretest	7,00	5,50; 7,00	4,00	2,00; 4,50	*p* = 0,499
		Posttest 1	5,00	3,00; 5,50			
		Posttest 2	3,00	1,00; 4,00			
	AHG	Pretest	7,00	5,00; 8,50	4,00	3,50; 5,00	
		Posttest 1	5,00	3,00; 6,00			
		Posttest 2	3,00	1,50; 4,50			
	Massage Therapy + AHG	Pretest	7,00	6,00; 7,50	4,00	4,00; 6,00	
		Posttest 1	6,00	4,50; 6,00			
		Posttest 2	3,00	0,50; 3,00			

Schober	Massage Therapy	Pretest	5,93	5,41; 6,38	0,83	0,20; 1,30	*p* = 0,256
		Posttest 1	6,52	6,04; 6,84			
		Posttest 2	6,76	6,01; 6,98			
	AHG	Pretest	6,53	5,53; 6,94	0,24	0,20; 0,58	
		Posttest 1	6,56	5,84; 7,28			
		Posttest 2	6,90	5,89; 7,45			
	Massage Therapy + AHG	Pretest	5,83	5,21; 6,01	0,60	0,33; 1,06	
		Posttest 1	6,16	5,73; 6,65			
		Posttest 2	6,26	5,81; 6,94			

SF-12	Massage Therapy	Pretest	32,00	31,00; 34,00	−1,00	−3,00; 1,00	*p* = 0,821
		Posttest 1	33,00	30,50; 34,00			
		Posttest 2	33,00	32,00; 34,50			
	AHG	Pretest	31,00	28,00; 33,00	−2,00	−4,00; 1,50	
		Posttest 1	32,00	29,00; 34,50			
		Posttest 2	31,00	30,00; 34,00			
	Massage Therapy + AHG	Pretest	32,00	29,00; 34,00	−3,00	−6,00; 2,50	
		Posttest 1	34,00	30,50; 36,00			
		Posttest 2	33,00	32,00; 34,50			

## Abbreviations

AGH: Abdominal Hypopressive Gymnastics
NRS: Numerical Rating Scale
ODI: Oswestry Disability Index
SD: Standard deviation.

## Additional Points

*Key Points*. (i) This is the first randomized controlled trial to analyze the effect of Abdominal Hypopressive Gymnastics (AHG) in subjects with chronic low back pain. (ii) Both therapies, massage and AHG, reduce pain, improve mobility, and reduce disability. (iii) Combined manual and active therapy seems to be more effective.
