# Corrigendum to “Effectiveness of Massage Therapy and Abdominal Hypopressive Gymnastics in Nonspecific Chronic Low Back Pain: A Randomized Controlled Pilot Study”

**DOI:** 10.1155/2018/3601984

**Published:** 2018-09-06

**Authors:** L. Bellido-Fernández, J. J. Jiménez-Rejano, R. Chillón-Martínez, M. A. Gómez-Benítez, M. De-La-Casa-Almeida, M. Rebollo-Salas

**Affiliations:** ^1^Physiotherapy Department, Faculty of Nursing, Physiotherapy and Podiatry, University of Seville, C/ Avicena S/N, 41009 Seville, Spain; ^2^Podiatry Department, University of Seville, C/ Avicena S/N, 41009 Seville, Spain

In the article titled “Effectiveness of Massage Therapy and Abdominal Hypopressive Gymnastics in Nonspecific Chronic Low Back Pain: A Randomized Controlled Pilot Study” [1], the corresponding author should be Dr. J. J. Jiménez-Rejano instead of Dr. L. Bellido-Fernández. In addition, there was an error in Figure 3 which included words in Spanish. The corrected figure is shown below.

## Figures and Tables

**Figure 3:**
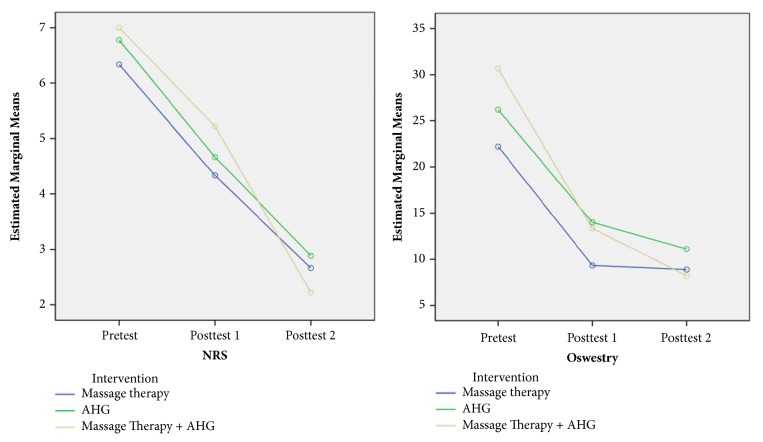
Marginal mean diagram of the Oswestry and NRS variable. The group receiving both treatments (Massage Therapy + AHG) obtained a greater statistically significant difference between pretest and posttest 2 against Massage Therapy group (*p *= 0,024).
